# Numerical Simulation of Maxillary Anterior Teeth Retraction Utilizing Power Arms in Lingual Orthodontic Technique

**DOI:** 10.3390/jpm14090988

**Published:** 2024-09-17

**Authors:** Shaher Alhiraky, Anna Konermann, Ludger Keilig, Christoph Bourauel

**Affiliations:** 1 Oral Technology, University Hospital Bonn, 53111 Bonn, Germany; 2 Department of Orthodontics, University Hospital Bonn, 53111 Bonn, Germany; 3 Department of Prosthetic Dentistry, Preclinical Education and Materials Science, University Hospital Bonn, 53111 Bonn, Germany

**Keywords:** bowing effect, en masse retraction, finite element method, lingual technique, power arm

## Abstract

Aims: It was the scope of this study to explore the biomechanical implications of retraction force application point modifications in lingual orthodontics, aiming to mitigate the bowing effect and enhance anchorage stability in the anterior teeth. Methods: Using the FE method on an idealized maxillary model, en masse retraction was simulated using a modified lingual fixed appliance including edgewise lingual brackets, a 0.017″ × 0.025″ mushroom-shaped archwire, and power arms between lateral incisors and canines, with a transpalatal arch (TPA) connecting the first molars. Applying bilateral retraction forces of 1.5 N at twelve positions, initial tooth displacements during space closure were evaluated. Results: Shifting power arms gingivally did not effectively counteract palatal tipping of incisors but reduced posterior and palatal tipping of canines with a power arm length of 11.3 mm preventing posterior tipping. Apically displacing the TPA retraction force increased mesiobuccal rotation while preventing mesial molar tipping for retraction forces applied 12.6 mm from the archwire. Conclusions: Apically shifting retraction forces can mitigate vertical bowing effects in lingual orthodontics, yet it also highlights the challenges in maintaining torque in the anterior teeth. Further research and clinical validation are essential in order to confirm these results, emphasizing the complexity and need for advanced biomechanical strategies in personalized lingual orthodontic treatments.

## 1. Introduction

The increasing emphasis on aesthetic demands in orthodontic treatment has substantially enhanced the popularity of lingual appliances due to their inconspicuous tooth correction. These invisible devices represent a major advancement in aesthetic orthodontics, further optimized by system improvements in terms of indirect lingual bracket bonding, innovative archwire materials, and computerized planning systems, thus increasing both the simplicity and precision of the technique [1,2]. The biomechanics of orthodontic tooth movement differs significantly between lingual and labial techniques due to the varying locations of force application relative to the center of resistance, resulting in distinct stress distributions and subsequent patterns of tooth displacement. Despite comparable efficacy to labial appliances, the specific biomechanical properties exhibited by lingual appliances necessitate modifications of traditional orthodontic mechanics to achieve optimal treatment outcomes [3,4].

With a prevalence of up to 84%, crowding is one of the most common malocclusions necessitating orthodontic treatment [5]. In contemporary orthodontic practice, space-gaining techniques encompass arch expansion, incisor proclination, interproximal reduction, molar distalization, molar uprighting, and derotation of the posterior teeth [6]. If these space-gaining techniques cannot be employed due to anatomical constraints or fail to generate sufficient space to address moderate to severe dental protrusion or crowding, tooth extraction may become necessary. In premolar extraction treatment, various strategies for space closure are available; however the lingual technique favors en masse retraction of the six anterior teeth [7]. This approach avoids separate full canine retraction, which could impede space closure due to the inherent requirement of using mushroom-shaped archwires with inset bends distal to the canine. These archwires are designed to accommodate the differences in labiolingual thickness between the anterior and posterior teeth. Additionally, this method addresses aesthetic concerns by preventing the formation of gaps between the lateral incisor and canine [2,3,8].

The two standard clinical procedures for en masse retraction imply loop mechanics utilizing a T-loop space closing spring to retract the anterior teeth directly and sliding mechanics involving an archwire guided by posterior brackets and tubes to move the anterior teeth collectively, facilitating bodily displacement [9,10]. En masse retraction techniques generally induce diverse tooth movements as side effects, implying bodily displacement, tipping, and associated changes in vertical and transversal dimensions throughout the dentition [11,12].

Several studies have demonstrated that lingual application of retraction forces can elicit a heightened bowing effect in both the vertical and horizontal dimensions compared to labial appliances [2,13,14]. The vertical bowing effect manifests as lingual tipping of the anterior teeth and, thus, torque loss, pre-contact between upper brackets and lower anterior teeth, mesial tipping of the posterior teeth, and posterior disclusion, whereas the transversal bowing effect creates mesiobuccal rotation of the molars and increases the inter-premolar distance [4,15].

To mitigate the bowing effect and prevent torque loss of the anterior teeth during space closure, phenomena that are more pronounced in lingual techniques and susceptible to significant vertical deviations, it is crucial to use a rigid archwire for space closure and to apply retraction forces that are lower than those typically used in labial appliances [3,4]. Addressing the challenge of incisor torque loss during lingual technique space closure may imply adjusting archwire torque, bracket positioning, or incorporating vertical compensating curves [2,4,14]. However, application of gable bends and compensating curves entail the drawback of potentially elevating friction between wire and brackets, thus complicating space closure [14,16].

Another promising approach to achieving effective torque control is the supplemental use of miniscrews, but finite element (FE) analyses revealed challenges such as initial lingual crown tipping and labial root tipping as well as occlusal crown extrusion, regardless of miniscrew height and lever arm position [7,17,18]. Consequently, while they can reduce some side effects, they are not able to ideally direct the force through the center of resistance.

Lever arm mechanics have been empirically validated as an efficacious approach for accomplishing en masse retraction of the anterior teeth while preventing undesirable torque loss of the front teeth. This technique relocates the line of action to align with the center of resistance, thereby enhancing the precision and effectiveness of the retraction process. In this context, the line of action corresponds to the position and orientation of the springs used to generate the retraction forces. A specific strategy to mitigate the unexpected bowing effect and maintain the arch form of the anterior six teeth during retraction involves placing the lever arms on the midpoint between lateral incisors and canines [7].

A variation in power arm length and the points of force application is likely to induce distinct movement patterns in the anterior teeth during the closure of extraction spaces, potentially facilitating or obstructing treatment objectives. Given that anchorage control is critical for achieving successful orthodontic outcomes, this study employed the FE method to analyze the use of different power arm lengths for the retraction force application and its impact on preventing the bowing effect. Therefore, initial movements and strain patterns in the periodontal ligament (PDL) of simulated teeth were investigated and compared across twelve different retraction force application positions in order to gain novel insights for the clinical application of the lingual technique. The aim of this study was to evaluate the impact of the position and orientation of springs employed to generate retraction forces on the rotational movement of the anterior teeth.

## 2. Material and Methods

The en masse retraction of maxillary anterior teeth was simulated using the FE method with the FE software system MSC Marc/Mentat (Marc/Mentat^®^ 2010, MSC Software Corp., Santa Ana, CA, USA). The FE model of the maxillary teeth and surrounding tooth-supporting structures—namely, PDL, alveolar bone, and gingiva—was generated based on a commercial three-dimensional data set (Digimation Corp., St Rose, LA, USA). To simulate a common extraction case, the first premolars were removed from this model, and the third molars were excluded to simplify the simulation. For the generation of a suitable lingual fixed appliance for the FE model, a mushroom-shaped lingual archwire with a rectangular cross-section of 0.43 × 0.65 mm^2^ (0.017″ × 0.025″) was designed to closely match the dental arch geometry. A power arm was attached to the archwire at the midpoints between the lateral incisors and canines in both the first and second quadrants. Additionally, two types of idealized lingual brackets were created, consisting of single brackets with a width at base of 2.2 mm for the six anterior teeth and twin brackets with a width at base of 2.9 mm for the posterior teeth, each featuring a slot width of 0.457 mm (0.018″).

**Figure 1:**
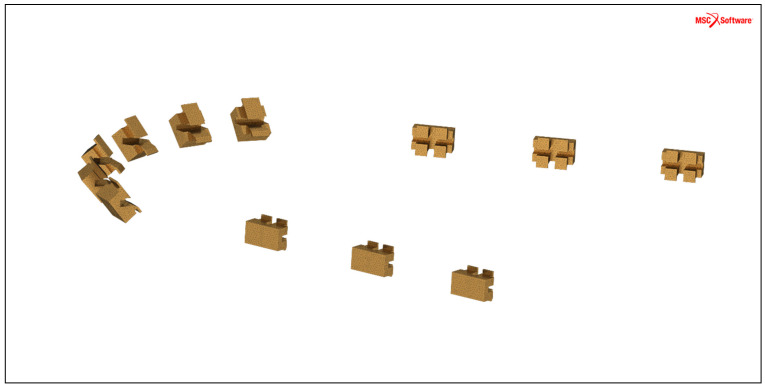
Design of lingual brackets for the FE simulated maxillary model.

**Figure 2:**
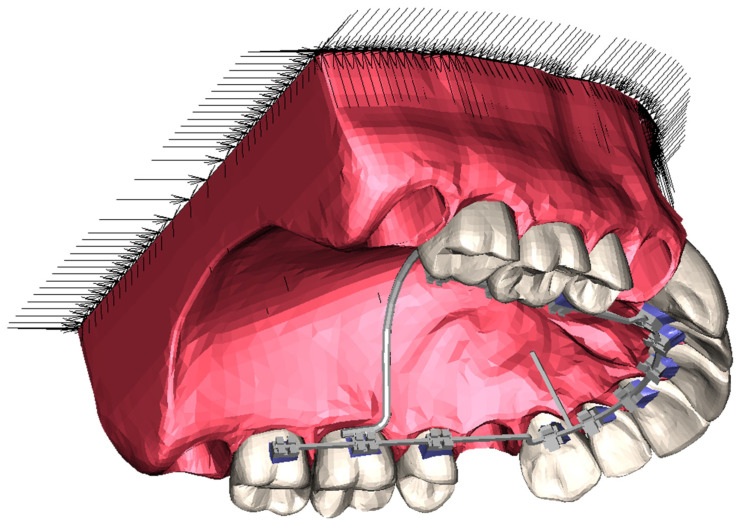
FE model of the simulated maxillary situation with extracted first premolars and a modified lingual fixed appliance consisting of self-modeled edgewise lingual brackets, a mushroom-shaped archwire with two power arms between the lateral incisors and canines, and a TPA. The nodes at the base of the model were constrained in all three translational directions, as indicated by the arrows.

After placement of the lingual archwire on the FE model, the lingual brackets were passively positioned in the center of the palatal surfaces of the maxillary teeth, the gaps between bracket bases and tooth palatal surfaces were filled with adhesive to customize the bracket bases, and a transpalatal arch (TPA) was applied to the first molars. The design of the lingual brackets and of the FE model are presented in Figure 1 and Figure 2, respectively.

The entire maxillary model, including the generated lingual fixed appliance, was comprised of approximately 137,000 nodes and 645,000 elements. This model utilized 6-noded pentahedral elements for the TPA, 8-noded hexahedral elements for the archwire, and 4-noded tetrahedral elements for the remaining components. The simulations were performed as nonlinear contact analyses.

Material parameters for the study were adopted from prior research (Table 1), assuming homogeneous and isotropic properties for all materials except for the PDL, which was represented as bilinear elastic [19,20]. Stainless steel properties were assigned uniformly to all components of the lingual appliance, including brackets, a lingual archwire with power arms, ligatures, and TPA.

**Figure 3:**
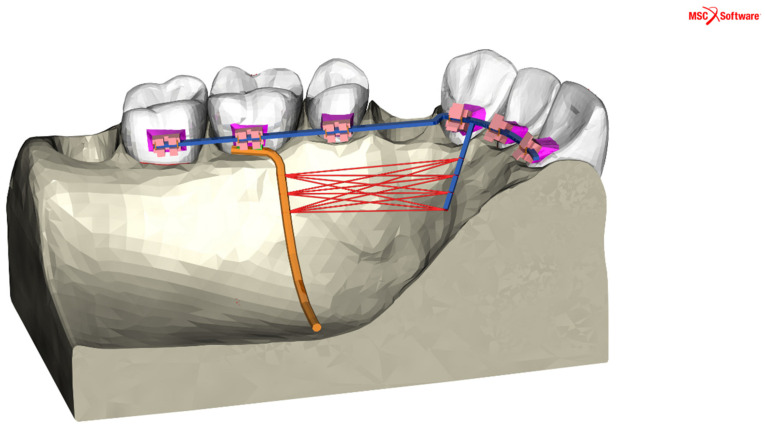
The twelve potential positions for the retraction springs represented by red lines, determined by the four points along the power arms (4, 6, 8, and 10 mm from the archwire) and the three points along the Transpalatal Arch (TPA) (4, 6, and 8 mm from the archwire) within the FE simulated maxillary model. Each simulation was conducted with only one activated spring, symmetrically positioned on both sides of the model.

Space closure was conducted using a 1.5 N retraction force applied symmetrically in both the first and second quadrants between the TPA and the power arms at various heights. The springs were strategically positioned at one of four specified locations along the power arms (at distances of 4, 6, 8, and 10 mm from the archwire) and one of three locations along the TPA (at distances of 4, 6, and 8 mm from the archwire). This arrangement yielded a total of twelve unique configurations for the retraction springs, as depicted in Figure 3. Separate simulations were conducted for each symmetrical spring configuration. Additionally, a boundary condition enforcing a fixed displacement of 0 mm along all three axes was applied to the maxillary bone base, serving as an anchor for the model during the simulation, as indicated by the arrows in Figure 2. The variation in spring configurations was determined by the geometric properties of the TPA and power arm, with a deliberate 2 mm step size chosen based on lever arm mechanics. In finite element analysis, where identical inputs yield identical outputs, the sample size effectively remains 1, as varying only the spring positions aligns with standard practices in numerical studies exploring specific parameter effects on a fixed model.

To determine the biomechanical effects of the different lines of action of retraction forces, the initial displacement of the crown and the root apex of each of the six anterior teeth and the first molars were determined in three-dimensional space using the global Cartesian coordinate system. In addition, the rotations of these teeth around the global coordinate axes were recorded, and stresses and strains in the PDL were calculated to better understand bone remodeling processes and tooth movements.

## 3. Results

**Figure 4:**
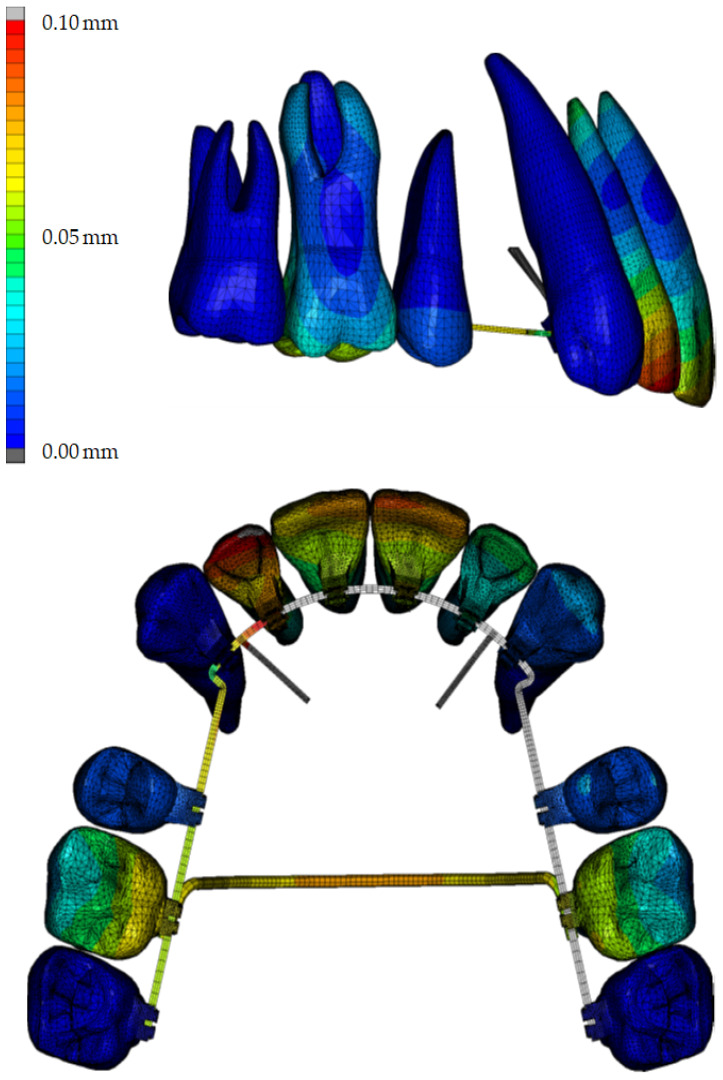
Example results of an en masse retraction simulation in the sagittal and transversal plane by visualizing the initial displacements of the teeth resulting from the acting forces with the help of color coding, with PDL and bone removed from the simulation for better visualization. The most significant movements measured in millimeters are highlighted in red, followed by yellow, green, and blue, in descending order of magnitude, as indicated by the legend on the left.

**Figure 5:**
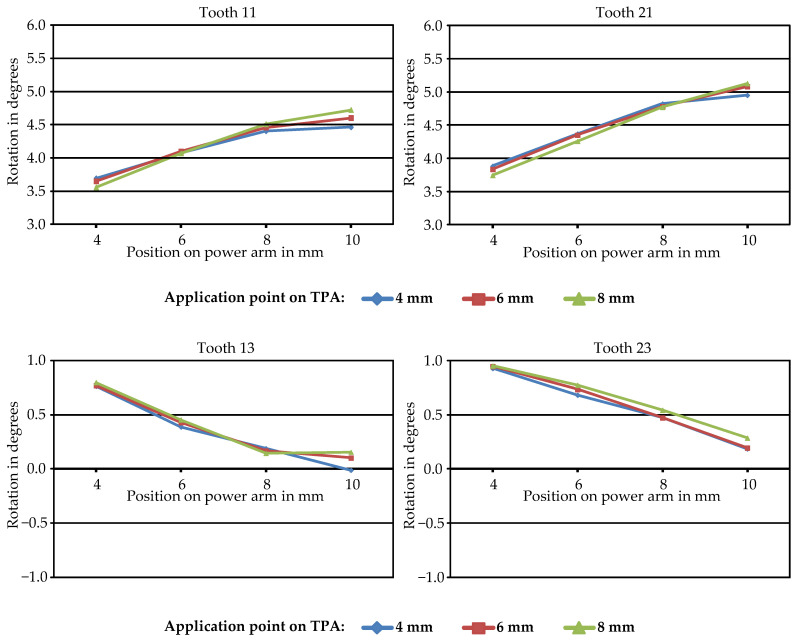
The rotation values in degrees (°) of teeth 11, 21 (upper row), 13, and 23 (lower row) around the *y*-axis plotted against the four different points of force application on the power arms (4 mm, 6 mm, 8 mm, and 10 mm) indicated on the *x*-axis. The results are depicted for three distinct force application points on the TPA, with corresponding color legends provided below the graphs. Each point represents an individual simulation.

Analysis of tooth movements indicated that all simulations demonstrated uncontrolled tipping of the central incisors, characterized by posterior and caudal movement of the tooth crowns, and anterior and cranial root movements. As visible in Figure 4, depicting the example outcomes of one simulation in both sagittal and transverse planes by visualizing the displacements resulting from applied forces using color coding as much as excluding PDL and bone, the central and lateral incisors exhibited greater displacement compared to the other teeth. Figure 5 (upper row) further illustrates that palatal tipping of the central incisors ranged from 3.6° to 5.1°. At this, the point of force application on the TPA had minimal impact on palatal tipping of the central incisors, whereas longer power arms resulted in a proportional increase in palatal tipping, averaging 4.5% per additional millimeter in length. The lateral incisors exhibited palatal tipping under all retraction force positions, with longer power arms resulting in slightly reduced tipping, while positions closer to the gingiva on the TPA led to increased tipping.

In contrast to the incisors, the effect of displacing the position of the springs used for the retraction forces on the movement pattern of the canines was very clear, particularly through adjustments in power arm length, which is shown in Figure 5 (lower row). With the shortest power arm in the simulations of 4 mm, the canine crowns moved distally medially and caudally. As the length of the power arm increased, the distal and palatal displacements of the canine crowns decreased, and, ultimately, with the longest power arm of 10 mm, distal tipping of the canines ceased while exhibiting a slight buccal movement instead. On average, each additional millimeter in the length of the power arm decreased the posterior tipping of the canines by 21.8%, with optimal bodily movement observed at 11.3 mm.

**Figure 6:**
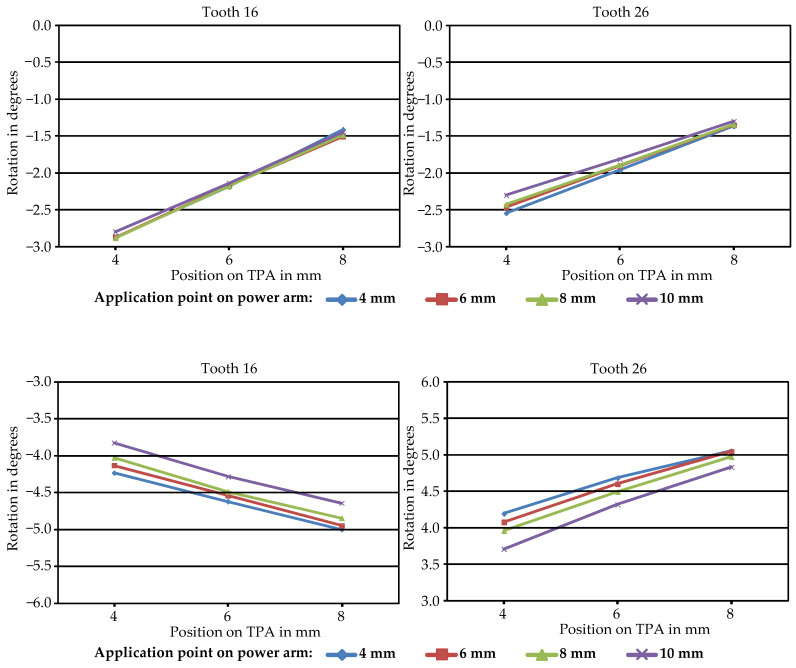
Rotation values in degrees (°) of teeth 16 and 26 for mesial tipping (upper row) and mesiobuccal rotation (lower row) plotted against the three different points of force application on the TPA (4 mm, 6 mm, and 8 mm) indicated on the *x*-axis. The results are depicted for four distinct points of force application on the power arms, with corresponding color legends provided below the graphs. Each point represents an individual simulation.

Simulations further revealed that the displacement of the springs significantly affected first molar behavior, whereas the point of force application on the TPA had a more substantial impact on first molar movement, and the length of the power arms having little effect. When retraction forces were applied 4 mm apically to the archwire on the TPA, the first molar crowns moved anteriorly, buccally, and caudally, with a mesiobuccal rotation. Increasing the apical displacement of the point of force application on the TPA reduced mesial tipping but increased mesiobuccal rotation. Specifically, each millimeter of apical displacement resulted in an average of 13.5% less mesial tipping and 5.4% more mesiobuccal rotation, which is presented in Figure 6. According to calculations based on these findings, retraction forces should be applied 12.6 mm away from the lingual archwire on the TPA to completely prevent mesial tipping of the first molars.

Table 2 presents the initial displacement along the three coordinate axes x, y, and z of the crowns as well as the root apices for all teeth investigated with retraction forces applied 8 mm apically to the archwire on the power arms and 6 mm apically on the TPA, and Table 3 documents the rotational adjustments of the teeth around these axes in response to these specified retraction forces.

Example results from the simulation with retraction forces applied 8 mm apically to the archwire on the power arms and 6 mm apically to the archwire on the TPA, illustrating initial displacements of crown and root apices of the investigated teeth along the three coordinate axes x, y, and z in micrometers (μm).

Given that strain induction in the PDL is the activating factor for biological changes and subsequent tooth movement, these strains were analyzed in this study for each condition in the FE simulations. Due to the elastic behavior of the PDL, the strain as a measure of the deformation in the PDL is better suited to show the mechanical reaction of this structure than the stresses in the same region. Regarding the distribution of strain within the PDL during simulated en masse retraction of the maxillary anterior teeth, analyses demonstrated that across all combinations of power arm and TPA force application points, the strain distributions in the PDL of the incisors were significantly higher compared to those in the PDL of the other teeth investigated. The lowest strain value for the PDL of the front teeth was 20.2% at a combination of a 10 mm power arm and 4 mm TPA point of force application, while the molar PDL exhibited a strain value of 16.9% under the same conditions. The highest strain value for the PDL of the anterior teeth was 24.2% but only 14.9% for molars at a combination of an 8 mm power arm and an 8 mm TPA point of force application.

**Figure 7:**
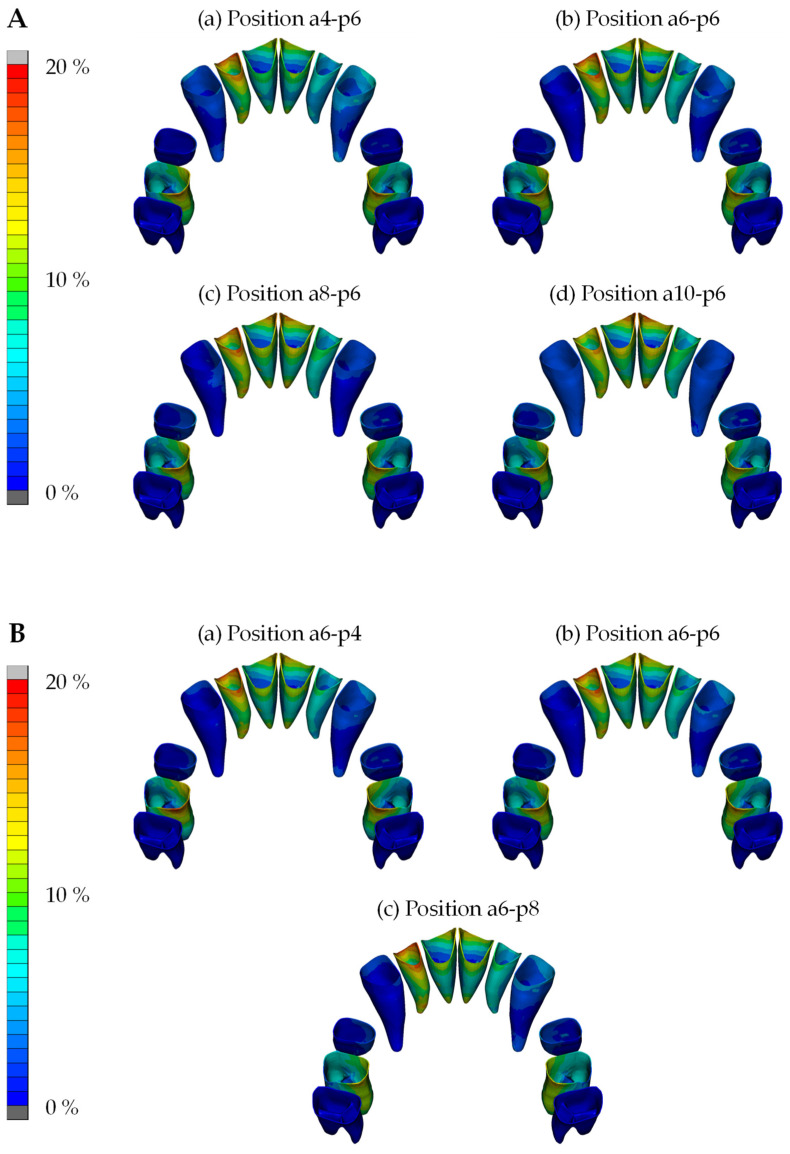
PDL strain distributions in four simulations with different power arm lengths (**A**) and in three simulations with different positions on the TPA (**B**). The most pronounced strain occurrences in the PDL (in %) are shown in yellow, followed by red color coding, as indicated in the legend on the left. In these diagrams, ‘a’ denotes the anterior teeth/front, and ‘p’ denotes the posterior teeth/molars. The numbers 4, 6, 8, and 10 represent the points of force application in millimeters.

The strain distributions are graphically illustrated and color-coded in Figure 7. Figure 7A depicts four simulations with different power arm lengths but the same TPA position, whereas Figure 7B shows three simulations with varying TPA positions but the same power arm length. Across all combinations, the strain distributions in the PDL of the incisors exhibited higher values and were less homogeneous compared to those in the PDL of other teeth. Notably, maximum strain values were observed at the root apices and alveolar crest, indicating a tendency for uncontrolled tipping of the incisors. In contrast, lengthening the power arms reduced the strains at the root apex and cervical region of the canines, as visible in Figure 7B, suggesting less initial tipping. Similarly, more apical force application on the TPA resulted in a more homogeneous strain distribution in the PDL of the first molars, indicating a tendency for bodily movement of the molars (Figure 7B).

## 4. Discussion

In this study, the biomechanical effects of modifying the position and orientation of springs used to generate retraction forces were investigated through FE analysis. This analysis focused on simulated en masse retraction of the six maxillary anterior teeth in a premolar extraction case employing a lingual appliance. The findings highlight increased tendencies towards an initial propensity for palatal tipping and loss of incisor torque, distal tipping of the canines, and mesial tipping accompanied by mesiobuccal rotation of the molars during space closure, which is consistent with previous research comparing the lingual approach to conventional vestibular appliances [2,4,21]. The results presented indicate that the application of retraction forces via power arms does not effectively prevent incisor torque loss during en masse retraction. Increasing power arm length up to 10 mm resulted in a slightly reduced palatal tipping of the lateral incisors but disadvantageously increased tipping of the central incisors. This aligns with prior research indicating that even 10 mm power arms between lateral incisors and canines were insufficient for achieving adequate torque control of the maxillary anterior teeth without additional measures [7].

Another significant finding in this study’s simulation outcomes was the decisive influence of the shift in the spring position and orientation on the initial movement of the canines. Although adjusting the force application points on the power arms or the TPA did not enhance torque control of the incisors, these modifications did affect canine movement. Specifically, the length of the power arms proved to be more critical than their posterior positioning on the TPA, with increased power arm length resulting in reduced distal and palatal tipping of the canines. With each additional millimeter in the length of the power arms, there was a 21.8% reduction in distal tipping of the canines. In simulations where the power arms were extended to 10 mm, posterior tipping of the right canine was completely eliminated, while the left canine exhibited minimal tipping of only 0.2°. The minor variations in tooth response between the two sides of the jaw in the simulation may be attributed to slight structural differences between the two halves of the FE model, variations in the manually adjusted lingual appliance, differing bracket positions, varying angles of the power arms, and potential prestress in the archwire. In contrast to our findings, another study revealed a tendency for the maxillary canines to exhibit labial crown tipping when the power arms were positioned between the lateral incisors and canines, together with lingual crown tipping when positioned distally to the canines [7]. Moreover, these tendencies were amplified with greater vertical distance between the points of force application on the power arm and the archwire plane. However, this study employed a different design by incorporating additional miniscrew anchorage, altering the overall force application system and thus making it not directly comparable to the one utilized in this work, thus providing a rationale for the observed discrepancies. Simulation results, moreover, highlighted that the position of force application had a significant impact on first molar behavior, with the point of force application on the TPA exerting a more substantial influence than the length of the power arms. Increasing the apical displacement of the force application point reduced mesial tipping but increased mesiobuccal rotation. Calculations performed on the basis of the findings suggest that to achieve optimal control of molar movement and prevent mesial molar tipping entirely, forces should be applied approximately 12.6 mm away from the lingual archwire on the TPA.

The current investigation undoubtedly possesses certain limitations necessitating thorough discussion. Prior investigations revealed that vertical positioning of the wire in the anterior slot, as typically employed in fully customized lingual appliances, is supposed to enhance torque control [22]. However, in this study, the horizontal placement of the wire in the anterior slot was simulated, which represents a widely adopted clinical practice found in many appliances. Moreover, it is important to note that retraction is usually executed in clinical practice using stable, larger dimensioned archwires, consistent with those utilized in this investigation. The selected archwire for simulations closely approached slot-filling dimensions, thereby minimizing disparities between the two systems, unlike the initial phase of treatment with undersized archwires where such variations might arise.

Another constraint of this study is that FE analysis is conducted in vitro, which does not adequately replicate the torsional behavior or play of the archwire within the bracket slot that occurs in vivo during clinical practice [7]. Furthermore, none of the models in this study were able to fully replicate the actual clinical conditions of periodontal structures. This limitation is due to the assumption of homogeneous properties in the model construction, which does not reflect the true heterogeneous nature of these structures in vivo. Despite this, computer-based three-dimensional numerical simulation remains the most effective method for simulating biomechanical forces and providing both qualitative and quantitative predictions of resultant tooth movements that closely approximate clinical outcomes [18].

A further significant limitation of the results concerns the clinical application of power arms in lingual orthodontic techniques. Many patients experience challenges in adapting to lingual components, raising concerns about patient acceptance, particularly when power arms exceed 10 mm in length. However, all lingual elements present potential interference, and some patients also show sensitivity to vestibular components. Therefore, individualized treatment planning is crucial, as it is impractical to universally include or exclude specific elements.

Moreover, this study only calculated the initial displacement of the teeth, which is a limitation because it does not account for the long-term biomechanical effects and progressive tooth movement that occur during the course of orthodontic treatment. Without this information, the findings may not fully capture the complexities of tooth movement dynamics or predict the final treatment outcomes accurately.

In summary, the results obtained with this FE simulation study have the potential of being a clinical contribution to improved and contemporary lingual appliance treatment planning, optimized system performance, and, thus, successful treatment outcomes and patient satisfaction. The findings revealed that the displacement of the springs generating retraction forces apically on both the TPA and power arms can influence the bodily movement of the canines and counteract the mesial tipping of the first molars. However, this approach does not effectively prevent mesiobuccal rotation of the first molars, nor does it sufficiently address the loss of torque in the maxillary incisors. Consequently, the technique of en masse retraction for space closure necessitates the implementation of supplementary methods for torque control. Future research directions emerging from our study should focus on the comprehensive simulation of tooth movement throughout the closure of extraction spaces. This approach entails segmenting the entire tooth movement process into a series of incremental steps, while precisely calculating the mechanical loads applied to the periodontium. Additionally, incorporating theories of bone remodeling to simulate the adaptive changes in the alveolar bone surrounding the tooth roots will be crucial for advancing the accuracy and efficacy of these simulations [23].

The clinical application of these simulation findings could improve orthodontic treatment planning by allowing for more precise adjustments to retraction forces and auxiliary tools. This may lead to enhanced predictability and efficiency in managing tooth movements during en masse retraction with lingual appliances. Future research could involve clinical trials to validate these simulation results in in vivo settings and to explore the long-term effects of various retraction strategies on tooth movement and periodontal health. Additionally, investigations into patient-specific adaptations and acceptance of lingual components with varying power arm lengths could provide valuable insights for optimizing treatment protocols and improving the overall patient experience.

## 5. Conclusions

In conclusion, this study utilized finite element analysis to investigate the biomechanical implications of altering the spring position and orientation used for force application during en masse retraction of maxillary anterior teeth in lingual orthodontics. Shifting the springs apically between the TPA and power arms failed to prevent maxillary incisor torque loss during retraction, necessitating alternate strategies to achieve adequate torque control. Increasing power arm length reduced distal and palatal tipping of the canines with optimal bodily movement occurring at a length of 11.3 mm. Apically positioning retraction forces on the TPA reduced mesial tipping of first molars, requiring forces applied 12.6 mm apically to the lingual archwire for complete prevention of tipping. However, this approach exacerbated mesiobuccal rotation of first molars, necessitating additional methods to mitigate rotation. Lastly, further finite element simulations and clinical studies are warranted to comprehensively validate these findings. The findings emphasize the complexity of achieving predictable outcomes in lingual orthodontic treatments and highlight the necessity for advanced biomechanical strategies and individualized treatment approaches.

## Figures and Tables

**Table 1:** Material parameters of the model structures utilized in the investigation.

Material	Young’s Modulus (MPa)	Poisson’s Ratio μ
Tooth (average value)	20,000	0.30
Bone (average value)	2000	0.30
Periodontal ligament	Bilinear, E1 = 0.05/E2 = 0.20 ε12: 7.0%	0.30
Lingual appliance (steel)	200,000	0.30

**Table 2:** Example results from the simulation with retraction forces.

Tooth		11	12	13	16	21	22	23	26
Crown	X	−77.8	−77.5	−0.3	21.9	−86.1	−35.7	−7.8	18.0
	Y	−5.3	55.0	−4.2	−11.3	−4.0	−33.8	−6.5	14.9
	Z	−12.4	−20.2	−5.3	5.1	−17.6	−2.5	−8.3	5.4
Root	X	29.9	31.5	1.9	−15.4	34.2	10.4	4.4	−13.0
	Y	5.3	−21.7	1.4	−6.2	0.0	14.3	3.0	6.2
	Z	21.2	32.3	−5.6	−0.2	21.8	23.0	−1.4	−0.1

**Table 3:** Initial rotation of the teeth around the coordinate axes x, y, and z in degrees (°) of the example simulation.

Tooth		11	12	13	16	21	22	23	26
	X	−1.0	2.7	−0.4	0.3	−0.1	−2.2	−0.3	−0.3
	Y	4.5	5.0	0.2	−2.2	4.8	1.9	0.5	−1.9
	Z	1.7	1.8	0.4	−4.4	−0.1	0.5	−0.1	4.5

## Data Availability

The original contributions presented in the study are included in the article, further inquiries can be directed to the corresponding author.
